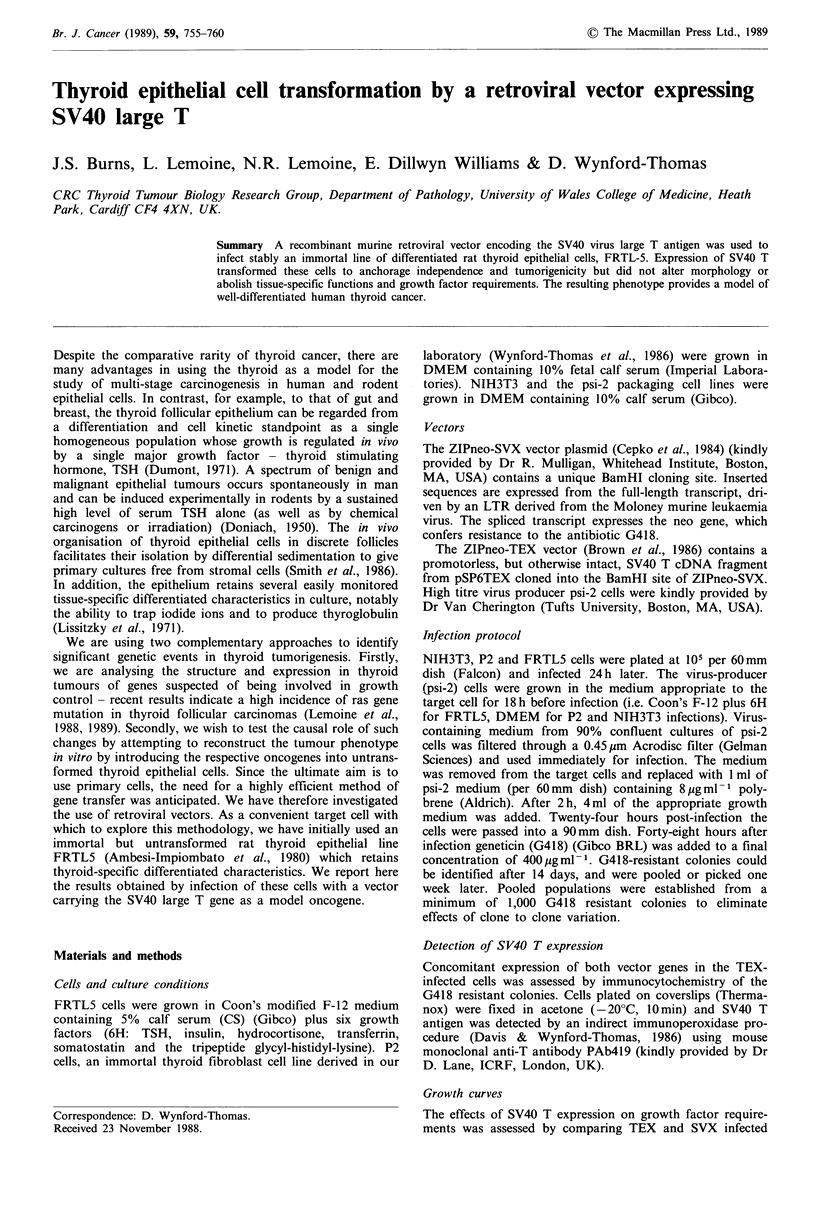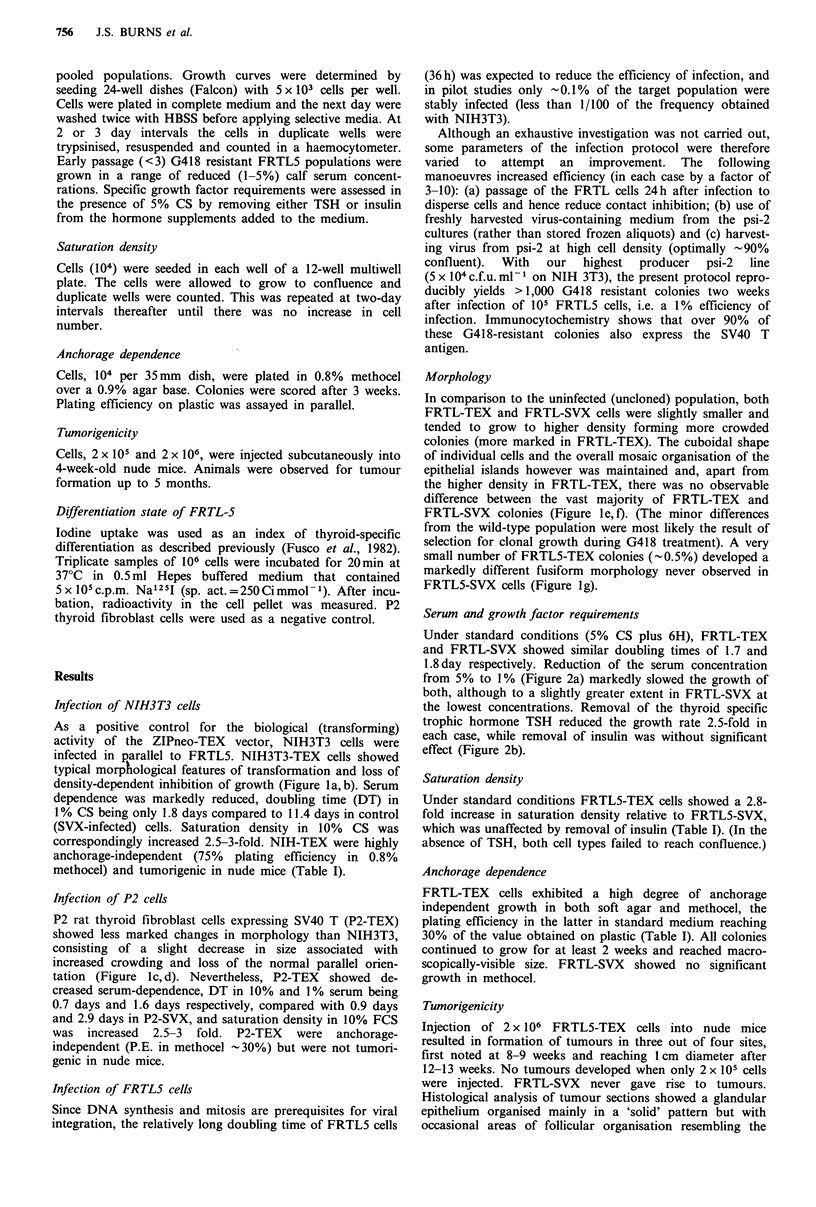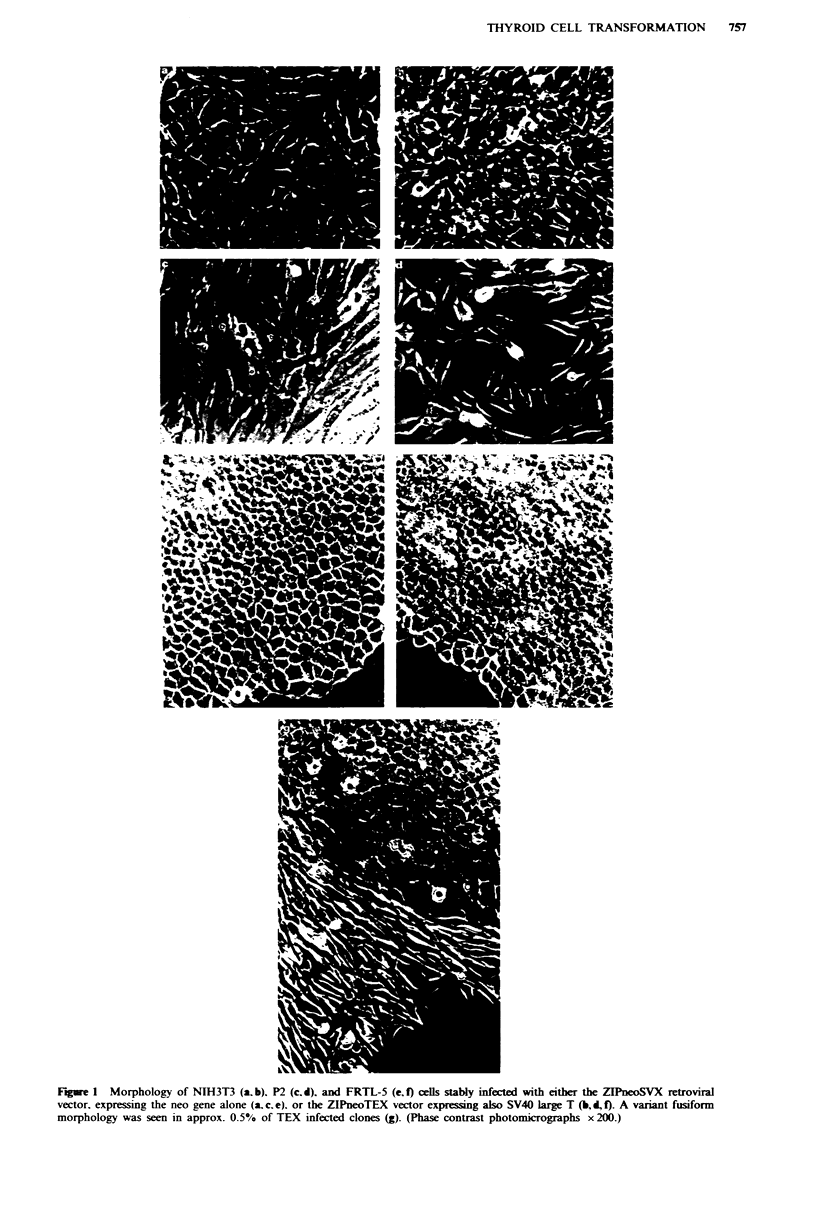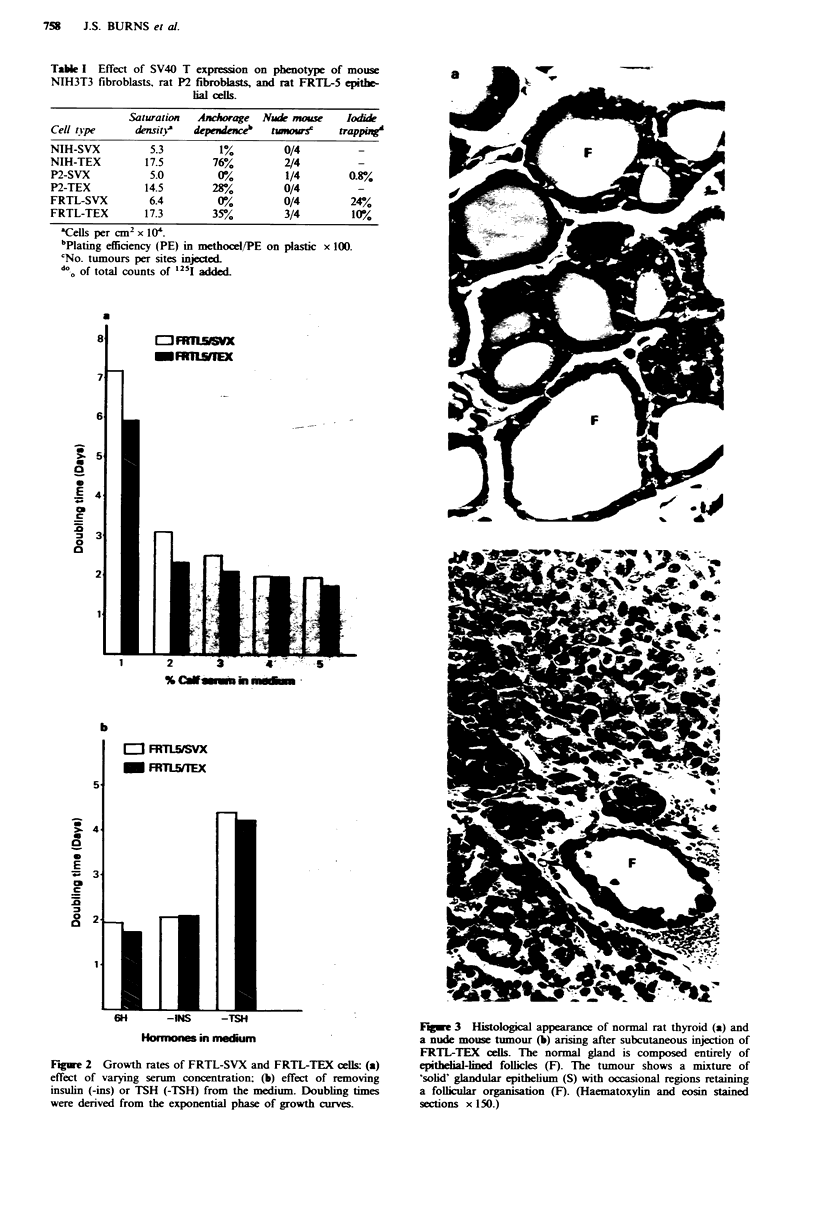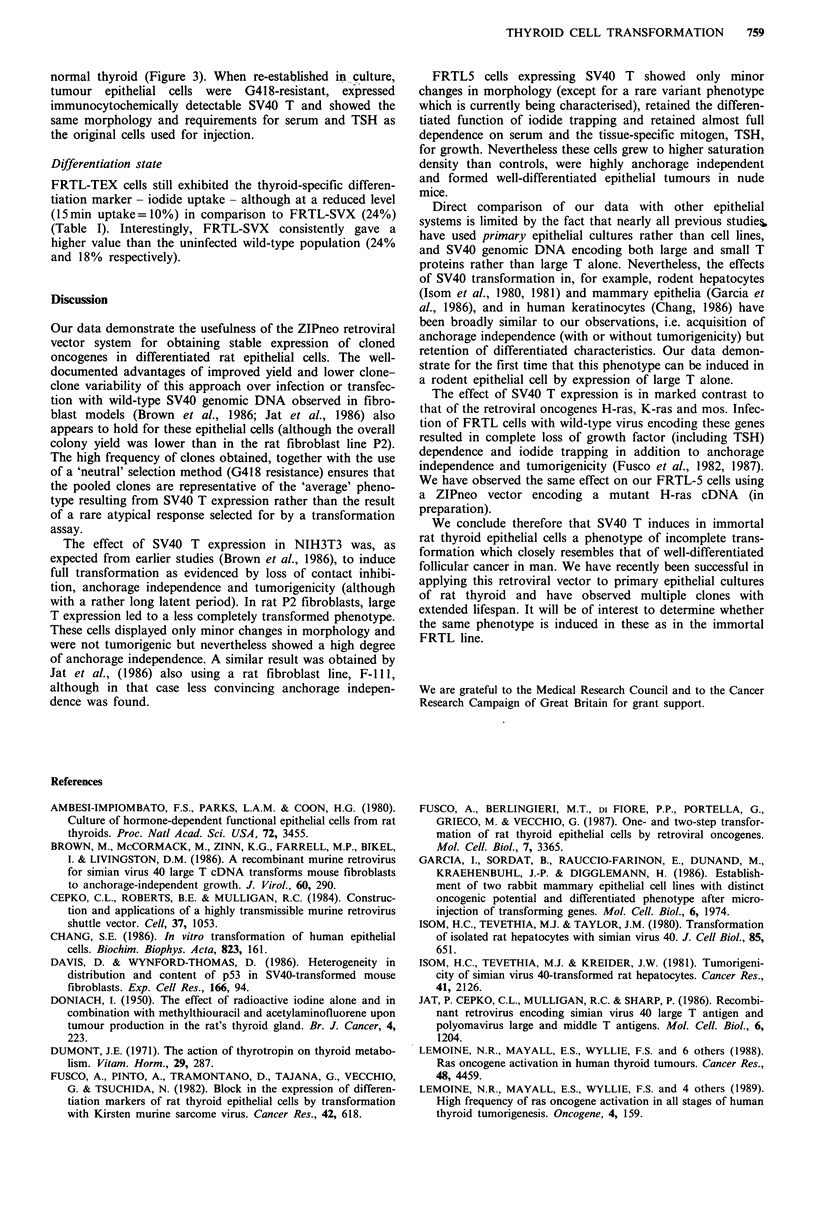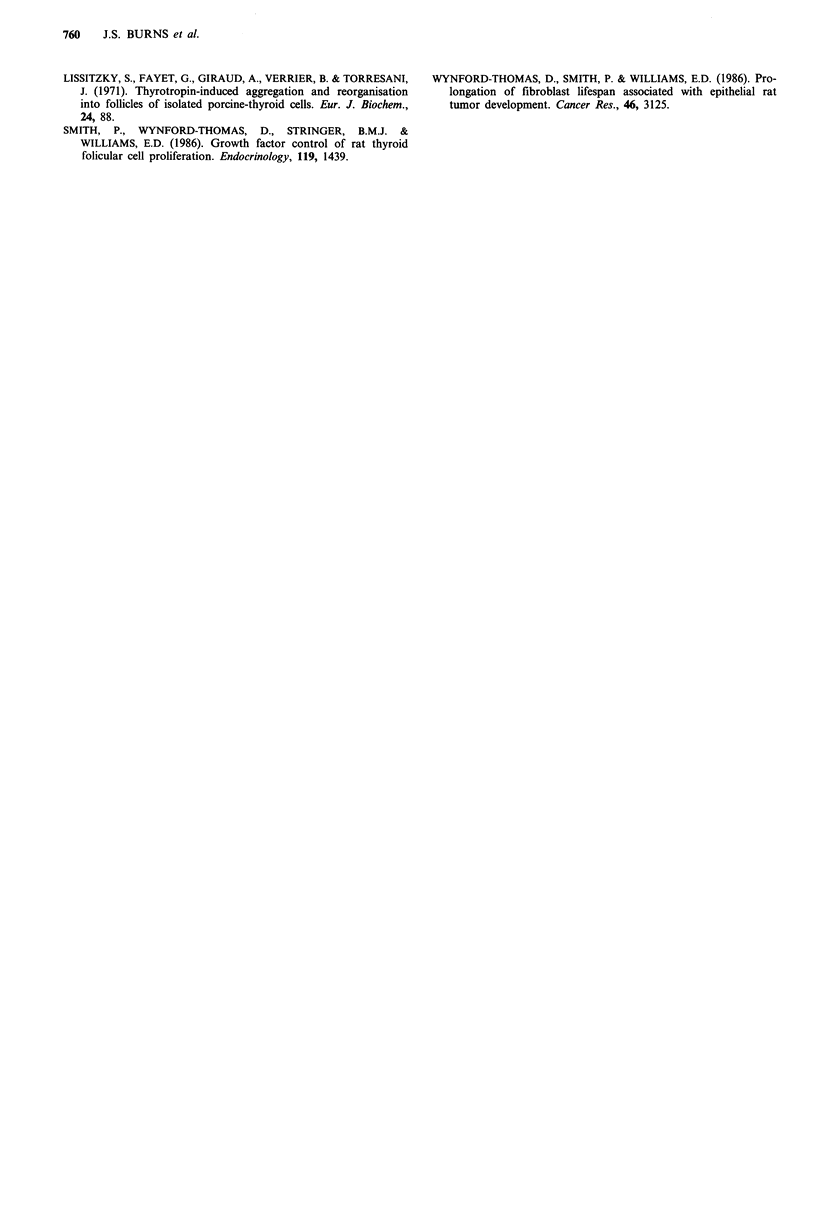# Thyroid epithelial cell transformation by a retroviral vector expressing SV40 large T.

**DOI:** 10.1038/bjc.1989.158

**Published:** 1989-05

**Authors:** J. S. Burns, L. Lemoine, N. R. Lemoine, E. D. Williams, D. Wynford-Thomas

**Affiliations:** Department of Pathology, University of Wales College of Medicine, Heath Park, Cardiff, UK.

## Abstract

**Images:**


*Br. J. Cancer* (1989), **59**, 755–760 © The Macmillan Press Ltd., 1989

### Thyroid epithelial cell transformation by a retroviral vector expressing SV40 large T

J.S. Burns, L. Lemoine, N.R. Lemoine, E. Dillwyn Williams & D. Wynford-Thomas

*CRC Thyroid Tumour Biology Research Group, Department of Pathology, University of Wales College of Medicine, Heath Park, Cardiff CF4 4XN, UK.*

**Summary** A recombinant murine retroviral vector encoding the SV40 virus large T antigen was used to infect stably an immortal line of differentiated rat thyroid epithelial cells, FRTL-5. Expression of SV40 T transformed these cells to anchorage independence and tumorigenicity but did not alter morphology or abolish tissue-specific functions and growth factor requirements. The resulting phenotype provides a model of well-differentiated human thyroid cancer.

Despite the comparative rarity of thyroid cancer, there are many advantages in using the thyroid as a model for the study of multi-stage carcinogenesis in human and rodent epithelial cells. In contrast, for example, to that of gut and breast, the thyroid follicular epithelium can be regarded from a differentiation and cell kinetic standpoint as a single homogeneous population whose growth is regulated *in vivo* by a single major growth factor – thyroid stimulating hormone, TSH (Dumont, 1971). A spectrum of benign and malignant epithelial tumours occurs spontaneously in man and can be induced experimentally in rodents by a sustained high level of serum TSH alone (as well as by chemical carcinogens or irradiation) (Doniach, 1950). The *in vivo* organisation of thyroid epithelial cells in discrete follicles facilitates their isolation by differential sedimentation to give primary cultures free from stromal cells (Smith *et al.*, 1986). In addition, the epithelium retains several easily monitored tissue-specific differentiated characteristics in culture, notably the ability to trap iodide ions and to produce thyroglobulin (Lissitzky *et al.*, 1971).

We are using two complementary approaches to identify significant genetic events in thyroid tumorigenesis. Firstly, we are analysing the structure and expression in thyroid tumours of genes suspected of being involved in growth control – recent results indicate a high incidence of ras gene mutation in thyroid follicular carcinomas (Lemoine *et al.*, 1988, 1989). Secondly, we wish to test the causal role of such changes by attempting to reconstruct the tumour phenotype *in vitro* by introducing the respective oncogenes into untransformed thyroid epithelial cells. Since the ultimate aim is to use primary cells, the need for a highly efficient method of gene transfer was anticipated. We have therefore investigated the use of retroviral vectors. As a convenient target cell with which to explore this methodology, we have initially used an immortal but untransformed rat thyroid epithelial line FRTL5 (Ambesi-Impiombato *et al.*, 1980) which retains thyroid-specific differentiated characteristics. We report here the results obtained by infection of these cells with a vector carrying the SV40 large T gene as a model oncogene.

### Materials and methods

### *Cells and culture conditions*

FRTL5 cells were grown in Coon's modified F-12 medium containing 5% calf serum (CS) (Gibco) plus six growth factors (6H: TSH, insulin, hydrocortisone, transferrin, somatostatin and the tripeptide glycyl-histidyl-lysine). P2 cells, an immortal thyroid fibroblast cell line derived in our laboratory (Wynford-Thomas *et al.*, 1986) were grown in DMEM containing 10% fetal calf serum (Imperial Laboratories). NIH3T3 and the psi-2 packaging cell lines were grown in DMEM containing 10% calf serum (Gibco).

### *Vectors*

The ZIPneo-SVX vector plasmid (Cepko *et al.*, 1984) (kindly provided by Dr R. Mulligan, Whitehead Institute, Boston, MA, USA) contains a unique BamHI cloning site. Inserted sequences are expressed from the full-length transcript, driven by an LTR derived from the Moloney murine leukaemia virus. The spliced transcript expresses the neo gene, which confers resistance to the antibiotic G418.

The ZIPneo-TEX vector (Brown *et al.*, 1986) contains a promotorless, but otherwise intact, SV40 T cDNA fragment from pSP6TEX cloned into the BamHI site of ZIPneo-SVX. High titre virus producer psi-2 cells were kindly provided by Dr Van Cherington (Tufts University, Boston, MA, USA).

### *Infection protocol*

NIH3T3, P2 and FRTL5 cells were plated at $10^5$ per 60 mm dish (Falcon) and infected 24 h later. The virus-producer (psi-2) cells were grown in the medium appropriate to the target cell for 18 h before infection (i.e. Coon's F-12 plus 6H for FRTL5, DMEM for P2 and NIH3T3 infections). Virus-containing medium from 90% confluent cultures of psi-2 cells was filtered through a 0.45 $\mu$m Acrodisc filter (Gelman Sciences) and used immediately for infection. The medium was removed from the target cells and replaced with 1 ml of psi-2 medium (per 60 mm dish) containing 8 $\mu g\,ml^{-1}$ polybrene (Aldrich). After 2 h, 4 ml of the appropriate growth medium was added. Twenty-four hours post-infection the cells were passed into a 90 mm dish. Forty-eight hours after infection geneticin (G418) (Gibco BRL) was added to a final concentration of 400 $\mu g\,ml^{-1}$. G418-resistant colonies could be identified after 14 days, and were pooled or picked one week later. Pooled populations were established from a minimum of 1,000 G418 resistant colonies to eliminate effects of clone to clone variation.

### *Detection of SV40 T expression*

Concomitant expression of both vector genes in the TEX-infected cells was assessed by immunocytochemistry of the G418 resistant colonies. Cells plated on coverslips (Thermanox) were fixed in acetone (−20°C, 10 min) and SV40 T antigen was detected by an indirect immunoperoxidase procedure (Davis & Wynford-Thomas, 1986) using mouse monoclonal anti-T antibody PAb419 (kindly provided by Dr D. Lane, ICRF, London, UK).

### *Growth curves*

The effects of SV40 T expression on growth factor requirements was assessed by comparing TEX and SVX infected

Correspondence: D. Wynford-Thomas.
Received 23 November 1988.

pooled populations. Growth curves were determined by seeding 24-well dishes (Falcon) with $5 \times 10^3$ cells per well. Cells were plated in complete medium and the next day were washed twice with HBSS before applying selective media. At 2 or 3 day intervals the cells in duplicate wells were trypsinised, resuspended and counted in a haemocytometer. Early passage (<3) G418 resistant FRTL5 populations were grown in a range of reduced (1–5%) calf serum concentrations. Specific growth factor requirements were assessed in the presence of 5% CS by removing either TSH or insulin from the hormone supplements added to the medium.

*Saturation density*

Cells ($10^4$) were seeded in each well of a 12-well multiwell plate. The cells were allowed to grow to confluence and duplicate wells were counted. This was repeated at two-day intervals thereafter until there was no increase in cell number.

*Anchorage dependence*

Cells, $10^4$ per 35 mm dish, were plated in 0.8% methocel over a 0.9% agar base. Colonies were scored after 3 weeks. Plating efficiency on plastic was assayed in parallel.

*Tumorigenicity*

Cells, $2 \times 10^5$ and $2 \times 10^6$, were injected subcutaneously into 4-week-old nude mice. Animals were observed for tumour formation up to 5 months.

*Differentiation state of FRTL-5*

Iodine uptake was used as an index of thyroid-specific differentiation as described previously (Fusco *et al.*, 1982). Triplicate samples of $10^6$ cells were incubated for 20 min at 37°C in 0.5 ml Hepes buffered medium that contained $5 \times 10^5$ c.p.m. $Na^{125}I$ (sp. act. = 250 Ci mmol$^{-1}$). After incubation, radioactivity in the cell pellet was measured. P2 thyroid fibroblast cells were used as a negative control.

### Results

*Infection of NIH3T3 cells*

As a positive control for the biological (transforming) activity of the ZIPneo-TEX vector, NIH3T3 cells were infected in parallel to FRTL5. NIH3T3-TEX cells showed typical morphological features of transformation and loss of density-dependent inhibition of growth (Figure 1a, b). Serum dependence was markedly reduced, doubling time (DT) in 1% CS being only 1.8 days compared to 11.4 days in control (SVX-infected) cells. Saturation density in 10% CS was correspondingly increased 2.5–3-fold. NIH-TEX were highly anchorage-independent (75% plating efficiency in 0.8% methocel) and tumorigenic in nude mice (Table I).

*Infection of P2 cells*

P2 rat thyroid fibroblast cells expressing SV40 T (P2-TEX) showed less marked changes in morphology than NIH3T3, consisting of a slight decrease in size associated with increased crowding and loss of the normal parallel orientation (Figure 1c, d). Nevertheless, P2-TEX showed decreased serum-dependence, DT in 10% and 1% serum being 0.7 days and 1.6 days respectively, compared with 0.9 days and 2.9 days in P2-SVX, and saturation density in 10% FCS was increased 2.5–3 fold. P2-TEX were anchorage-independent (P.E. in methocel ~30%) but were not tumorigenic in nude mice.

*Infection of FRTL5 cells*

Since DNA synthesis and mitosis are prerequisites for viral integration, the relatively long doubling time of FRTL5 cells (36 h) was expected to reduce the efficiency of infection, and in pilot studies only ~0.1% of the target population were stably infected (less than 1/100 of the frequency obtained with NIH3T3).

Although an exhaustive investigation was not carried out, some parameters of the infection protocol were therefore varied to attempt an improvement. The following manoeuvres increased efficiency (in each case by a factor of 3–10): (a) passage of the FRTL cells 24 h after infection to disperse cells and hence reduce contact inhibition; (b) use of freshly harvested virus-containing medium from the psi-2 cultures (rather than stored frozen aliquots) and (c) harvesting virus from psi-2 at high cell density (optimally ~90% confluent). With our highest producer psi-2 line ($5 \times 10^4$ c.f.u. ml$^{-1}$ on NIH 3T3), the present protocol reproducibly yields >1,000 G418 resistant colonies two weeks after infection of $10^5$ FRTL5 cells, i.e. a 1% efficiency of infection. Immunocytochemistry shows that over 90% of these G418-resistant colonies also express the SV40 T antigen.

*Morphology*

In comparison to the uninfected (uncloned) population, both FRTL-TEX and FRTL-SVX cells were slightly smaller and tended to grow to higher density forming more crowded colonies (more marked in FRTL-TEX). The cuboidal shape of individual cells and the overall mosaic organisation of the epithelial islands however was maintained and, apart from the higher density in FRTL-TEX, there was no observable difference between the vast majority of FRTL-TEX and FRTL-SVX colonies (Figure 1e, f). (The minor differences from the wild-type population were most likely the result of selection for clonal growth during G418 treatment). A very small number of FRTL5-TEX colonies (~0.5%) developed a markedly different fusiform morphology never observed in FRTL5-SVX cells (Figure 1g).

*Serum and growth factor requirements*

Under standard conditions (5% CS plus 6H), FRTL-TEX and FRTL-SVX showed similar doubling times of 1.7 and 1.8 day respectively. Reduction of the serum concentration from 5% to 1% (Figure 2a) markedly slowed the growth of both, although to a slightly greater extent in FRTL-SVX at the lowest concentrations. Removal of the thyroid specific trophic hormone TSH reduced the growth rate 2.5-fold in each case, while removal of insulin was without significant effect (Figure 2b).

*Saturation density*

Under standard conditions FRTL5-TEX cells showed a 2.8-fold increase in saturation density relative to FRTL5-SVX, which was unaffected by removal of insulin (Table I). (In the absence of TSH, both cell types failed to reach confluence.)

*Anchorage dependence*

FRTL-TEX cells exhibited a high degree of anchorage independent growth in both soft agar and methocel, the plating efficiency in the latter in standard medium reaching 30% of the value obtained on plastic (Table I). All colonies continued to grow for at least 2 weeks and reached macroscopically-visible size. FRTL-SVX showed no significant growth in methocel.

*Tumorigenicity*

Injection of $2 \times 10^6$ FRTL5-TEX cells into nude mice resulted in formation of tumours in three out of four sites, first noted at 8–9 weeks and reaching 1 cm diameter after 12–13 weeks. No tumours developed when only $2 \times 10^5$ cells were injected. FRTL-SVX never gave rise to tumours. Histological analysis of tumour sections showed a glandular epithelium organised mainly in a 'solid' pattern but with occasional areas of follicular organisation resembling the

**Figure 1** Morphology of NIH3T3 (**a**, **b**), P2 (**c**, **d**), and FRTL-5 (**e**, **f**) cells stably infected with either the ZIPneoSVX retroviral vector, expressing the neo gene alone (**a**, **c**, **e**), or the ZIPneoTEX vector expressing also SV40 large T (**b**, **d**, **f**). A variant fusiform morphology was seen in approx. 0.5% of TEX infected clones (**g**). (Phase contrast photomicrographs ×200.)

**Table I** Effect of SV40 T expression on phenotype of mouse NIH3T3 fibroblasts, rat P2 fibroblasts, and rat FRTL-5 epithelial cells.

| *Cell type* | *Saturation density*[a] | *Anchorage dependence*[b] | *Nude mouse tumours*[c] | *Iodide trapping*[d] |
|---|---|---|---|---|
| NIH-SVX | 5.3 | 1% | 0/4 | - |
| NIH-TEX | 17.5 | 76% | 2/4 | - |
| P2-SVX | 5.0 | 0% | 1/4 | 0.8% |
| P2-TEX | 14.5 | 28% | 0/4 | - |
| FRTL-SVX | 6.4 | 0% | 0/4 | 24% |
| FRTL-TEX | 17.3 | 35% | 3/4 | 10% |

[a]Cells per $cm^2 \times 10^4$.

[b]Plating efficiency (PE) in methocel/PE on plastic × 100.

[c]No. tumours per sites injected.

[d]% of total counts of $^{125}I$ added.

**Figure 2** Growth rates of FRTL-SVX and FRTL-TEX cells: (**a**) effect of varying serum concentration; (**b**) effect of removing insulin (-ins) or TSH (-TSH) from the medium. Doubling times were derived from the exponential phase of growth curves.

**Figure 3** Histological appearance of normal rat thyroid (**a**) and a nude mouse tumour (**b**) arising after subcutaneous injection of FRTL-TEX cells. The normal gland is composed entirely of epithelial-lined follicles (F). The tumour shows a mixture of 'solid' glandular epithelium (S) with occasional regions retaining a follicular organisation (F). (Haematoxylin and eosin stained sections × 150.)

normal thyroid (Figure 3). When re-established in culture, tumour epithelial cells were G418-resistant, expressed immunocytochemically detectable SV40 T and showed the same morphology and requirements for serum and TSH as the original cells used for injection.

*Differentiation state*

FRTL-TEX cells still exhibited the thyroid-specific differentiation marker – iodide uptake – although at a reduced level (15 min uptake = 10%) in comparison to FRTL-SVX (24%) (Table I). Interestingly, FRTL-SVX consistently gave a higher value than the uninfected wild-type population (24% and 18% respectively).

### Discussion

Our data demonstrate the usefulness of the ZIPneo retroviral vector system for obtaining stable expression of cloned oncogenes in differentiated rat epithelial cells. The well-documented advantages of improved yield and lower clone–clone variability of this approach over infection or transfection with wild-type SV40 genomic DNA observed in fibroblast models (Brown *et al.*, 1986; Jat *et al.*, 1986) also appears to hold for these epithelial cells (although the overall colony yield was lower than in the rat fibroblast line P2). The high frequency of clones obtained, together with the use of a 'neutral' selection method (G418 resistance) ensures that the pooled clones are representative of the 'average' phenotype resulting from SV40 T expression rather than the result of a rare atypical response selected for by a transformation assay.

The effect of SV40 T expression in NIH3T3 was, as expected from earlier studies (Brown *et al.*, 1986), to induce full transformation as evidenced by loss of contact inhibition, anchorage independence and tumorigenicity (although with a rather long latent period). In rat P2 fibroblasts, large T expression led to a less completely transformed phenotype. These cells displayed only minor changes in morphology and were not tumorigenic but nevertheless showed a high degree of anchorage independence. A similar result was obtained by Jat *et al.*, (1986) also using a rat fibroblast line, F-111, although in that case less convincing anchorage independence was found.

FRTL5 cells expressing SV40 T showed only minor changes in morphology (except for a rare variant phenotype which is currently being characterised), retained the differentiated function of iodide trapping and retained almost full dependence on serum and the tissue-specific mitogen, TSH, for growth. Nevertheless these cells grew to higher saturation density than controls, were highly anchorage independent and formed well-differentiated epithelial tumours in nude mice.

Direct comparison of our data with other epithelial systems is limited by the fact that nearly all previous studies have used *primary* epithelial cultures rather than cell lines, and SV40 genomic DNA encoding both large and small T proteins rather than large T alone. Nevertheless, the effects of SV40 transformation in, for example, rodent hepatocytes (Isom *et al.*, 1980, 1981) and mammary epithelia (Garcia *et al.*, 1986), and in human keratinocytes (Chang, 1986) have been broadly similar to our observations, i.e. acquisition of anchorage independence (with or without tumorigenicity) but retention of differentiated characteristics. Our data demonstrate for the first time that this phenotype can be induced in a rodent epithelial cell by expression of large T alone.

The effect of SV40 T expression is in marked contrast to that of the retroviral oncogenes H-ras, K-ras and mos. Infection of FRTL cells with wild-type virus encoding these genes resulted in complete loss of growth factor (including TSH) dependence and iodide trapping in addition to anchorage independence and tumorigenicity (Fusco *et al.*, 1982, 1987). We have observed the same effect on our FRTL-5 cells using a ZIPneo vector encoding a mutant H-ras cDNA (in preparation).

We conclude therefore that SV40 T induces in immortal rat thyroid epithelial cells a phenotype of incomplete transformation which closely resembles that of well-differentiated follicular cancer in man. We have recently been successful in applying this retroviral vector to primary epithelial cultures of rat thyroid and have observed multiple clones with extended lifespan. It will be of interest to determine whether the same phenotype is induced in these as in the immortal FRTL line.

We are grateful to the Medical Research Council and to the Cancer Research Campaign of Great Britain for grant support.

### References

AMBESI-IMPIOMBATO, F.S., PARKS, L.A.M. & COON, H.G. (1980). Culture of hormone-dependent functional epithelial cells from rat thyroids. *Proc. Natl Acad. Sci. USA*, **72**, 3455.

BROWN, M., McCORMACK, M., ZINN, K.G., FARRELL, M.P., BIKEL, I. & LIVINGSTON, D.M. (1986). A recombinant murine retrovirus for simian virus 40 large T cDNA transforms mouse fibroblasts to anchorage-independent growth. *J. Virol.*, **60**, 290.

CEPKO, C.L., ROBERTS, B.E. & MULLIGAN, R.C. (1984). Construction and applications of a highly transmissible murine retrovirus shuttle vector. *Cell*, **37**, 1053.

CHANG, S.E. (1986). *In vitro* transformation of human epithelial cells. *Biochim. Biophys. Acta*, **823**, 161.

DAVIS, D. & WYNFORD-THOMAS, D. (1986). Heterogeneity in distribution and content of p53 in SV40-transformed mouse fibroblasts. *Exp. Cell Res.*, **166**, 94.

DONIACH, I. (1950). The effect of radioactive iodine alone and in combination with methylthiouracil and acetylaminofluorene upon tumour production in the rat's thyroid gland. *Br. J. Cancer*, **4**, 223.

DUMONT, J.E. (1971). The action of thyrotropin on thyroid metabolism. *Vitam. Horm.*, **29**, 287.

FUSCO, A., PINTO, A., TRAMONTANO, D., TAJANA, G., VECCHIO, G. & TSUCHIDA, N. (1982). Block in the expression of differentiation markers of rat thyroid epithelial cells by transformation with Kirsten murine sarcome virus. *Cancer Res.*, **42**, 618.

FUSCO, A., BERLINGIERI, M.T., DI FIORE, P.P., PORTELLA, G., GRIECO, M. & VECCHIO, G. (1987). One- and two-step transformation of rat thyroid epithelial cells by retroviral oncogenes. *Mol. Cell. Biol.*, **7**, 3365.

GARCIA, I., SORDAT, B., RAUCCIO-FARINON, E., DUNAND, M., KRAEHENBUHL, J.-P. & DIGGLEMANN, H. (1986). Establishment of two rabbit mammary epithelial cell lines with distinct oncogenic potential and differentiated phenotype after microinjection of transforming genes. *Mol. Cell. Biol.*, **6**, 1974.

ISOM, H.C., TEVETHIA, M.J. & TAYLOR, J.M. (1980). Transformation of isolated rat hepatocytes with simian virus 40. *J. Cell Biol.*, **85**, 651.

ISOM, H.C., TEVETHIA, M.J. & KREIDER, J.W. (1981). Tumorigenicity of simian virus 40-transformed rat hepatocytes. *Cancer Res.*, **41**, 2126.

JAT, P. CEPKO, C.L., MULLIGAN, R.C. & SHARP, P. (1986). Recombinant retrovirus encoding simian virus 40 large T antigen and polyomavirus large and middle T antigens. *Mol. Cell. Biol.*, **6**, 1204.

LEMOINE, N.R., MAYALL, E.S., WYLLIE, F.S. and 6 others (1988). Ras oncogene activation in human thyroid tumours. *Cancer Res.*, **48**, 4459.

LEMOINE, N.R., MAYALL, E.S., WYLLIE, F.S. and 4 others (1989). High frequency of ras oncogene activation in all stages of human thyroid tumorigenesis. *Oncogene*, **4**, 159.

LISSITZKY, S., FAYET, G., GIRAUD, A., VERRIER, B. & TORRESANI, J. (1971). Thyrotropin-induced aggregation and reorganisation into follicles of isolated porcine-thyroid cells. *Eur. J. Biochem.*, **24,** 88.

SMITH, P., WYNFORD-THOMAS, D., STRINGER, B.M.J. & WILLIAMS, E.D. (1986). Growth factor control of rat thyroid folicular cell proliferation. *Endocrinology*, **119,** 1439.

WYNFORD-THOMAS, D., SMITH, P. & WILLIAMS, E.D. (1986). Prolongation of fibroblast lifespan associated with epithelial rat tumor development. *Cancer Res.*, **46,** 3125.